# Understanding the relationships between the physical environment and physical activity in older adults: a systematic review of qualitative studies

**DOI:** 10.1186/1479-5868-11-79

**Published:** 2014-07-17

**Authors:** Mika Moran, Jelle Van Cauwenberg, Rachel Hercky-Linnewiel, Ester Cerin, Benedicte Deforche, Pnina Plaut

**Affiliations:** 1 Faculty of Architecture and Town Planning, Technion – Israel Institute of Technology, Haifa 32000, Israel; 2 School of Public Health, Faculty of Social Welfare and Health Sciences, University of Haifa, Mount Carmel 31905, Israel; 3 Department of Human Biometry and Biomechanics, Faculty of Physical Education and Physical Therapy, Vrije Universiteit Brussel, Pleinlaan 2, B-1050 Brussel, Belgium; 4 Department of Movement and Sport Sciences, Faculty of Medicine and Health Sciences, Ghent University, Watersportlaan 2, B-9000 Ghent, Belgium; 5 Fund for Scientific Research Flanders (FWO), Egmontstraat 5, B-1000 Brussels, Belgium; 6 Institute of Human Performance, The University of Hong Kong, Pokfulam, Hong Kong; 7 Center of Physical Activity and Exercise Research, School of Exercise and Nutrition Sciences, Deakin University, Burwood, VIC, Australia

**Keywords:** Physical environment, Physical activity, Older adults, Qualitative research, Systematic review

## Abstract

**Background:**

While physical activity (PA) provides many physical, social, and mental health benefits for older adults, they are the least physically active age group. Ecological models highlight the importance of the physical environment in promoting PA. However, results of previous quantitative research revealed inconsistencies in environmental correlates of older adults’ PA that may be explained by methodological issues. Qualitative studies can inform and complement quantitative research on environment-PA relationships by providing insight into how and why the environment influences participants’ PA behaviors. The current study aimed to provide a systematic review of qualitative studies exploring the potential impact of the physical environment on older adults’ PA behaviors.

**Methods:**

A systematic search was conducted in databases of various disciplines, including: health, architecture and urban planning, transportation, and interdisciplinary databases. From 3,047 articles identified in the physical activity, initial search, 31 articles published from 1996 to 2012 met all inclusion criteria. An inductive content analysis was performed on the extracted findings to identify emerging environmental elements related to older adults’ PA. The identified environmental elements were then grouped by study methodologies [indoor interviews (individual or focus groups) vs spatial methods (photo-voice, observations, walk-along interviews)].

**Results:**

This review provides detailed information about environmental factors that potentially influence older adults’ PA behaviors. These factors were categorized into five themes: pedestrian infrastructure, safety, access to amenities, aesthetics, and environmental conditions. Environmental factors especially relevant to older adults (i.e., access to facilities, green open spaces and rest areas) tended to emerge more frequently in studies that combined interviews with spatial qualitative methods.

**Conclusions:**

Findings showed that qualitative research can provide in-depth information on environmental elements that influence older adults’ PA. Future qualitative studies on the physical environment and older adults’ PA would benefit from combining interviews with more spatially-oriented methods. Multidisciplinary mixed-methods studies are recommended to establish quantitative relationships complemented with in-depth qualitative information.

## Introduction

Older adults (≥ 65 years) are the fastest growing age segment of the western world population [1]. While physical activity (PA) provides many physical, social, and mental health benefits for older adults [2], they are the least physically active age group. In western countries, only 30-40% of those aged 65 years and older comply with the recommended 30 minutes of moderate-to-vigorous PA on at least five days/week [3,4]. In order to preserve older adults’ quality of life and manage health care costs, the promotion of PA in this age group is warranted [5,6].

To promote PA, social ecological models emphasize the need for multilevel interventions in which PA-stimulating physical environments are provided [7,8]. The physical environment encompasses the objective and perceived characteristics of the physical context in which people spend their time (e.g., home, neighborhood), including aspects of urban design (e.g., presence of sidewalks), traffic volume and speed, distance to and design of venues for PA (e.g., parks), and crime and safety [9]. Although the physical environment is considered to be especially relevant for older adults’ PA [10], environment-PA relationships are less frequently studied in older adults than in younger age groups [11].

Previous quantitative studies agreed upon the positive relationship between presence of nearby destinations and older adults’ walking for transportation [12-14]. However, a recent systematic review of quantitative studies [15] revealed inconsistencies in findings regarding other environmental correlates of older adults’ PA (e.g. quality of sidewalks, access to parks, availability of sport facilities, etc). These inconsistencies might be explained by methodological limitations inherent to the quantitative methods used [15]. Qualitative methods can address some of these limitations and carry the potential to inform and complement quantitative research on environment-PA relationships [15,16]. Qualitative methods use interactive strategies to understand the meanings of people’s interactions with their environments [17,18]. Consequently, these methods can help to explain not only what, but also how and why environmental factors relate to PA [7].

Qualitative research methods may include individual interviews, focus group discussions and spatially-oriented methods (e.g., on-site observation, photo-voice methodology, walk-along interviews). Qualitative individual interviews (either semi-structured or in-depth interviews) consist of open-ended questions that define an area to be explored in detail by the interviewees’ answers [19]. Focus group discussions benefit from group interactions that enable participants to explore their views and thereby highlight cultural values or group norms that are less accessible in individual interviews [20]. Qualitative spatial methods are claimed to help contextualize participants’ perceptions and experiences within their daily environment and, hence, may be particularly useful when exploring environmental perceptions and spatial behavior such as PA behaviors [21,22]. However, it is not clear whether qualitative spatial methods yield different and/or more detailed findings than indoor individual or focus group interviews.

The current study aims to provide a systematic review of qualitative studies exploring the potential impact of the physical environment on older adults’ PA behaviors. More specifically, we aim to (1) describe the characteristics and methodologies of qualitative studies conducted in this field, (2) identify recurring physical environmental themes and factors possibly related to older adults’ PA behaviors, and (3) compare the emerging themes and factors according to the qualitative method used (i.e., interview versus spatial qualitative methods).

## Methods

Guided by the PRISMA statement, we conducted a systematic and comprehensive search in several electronic databases. Inclusion and exclusion criteria were defined prior to this systematic search. These criteria were applied throughout the consecutive screenings for eligibility by title, abstract, and full text. For all articles included by full text, a back- and forward tracking procedure was performed to identify additional relevant articles. Searches were conducted independently by MM, JVC, RH, PP and EC. In case of any doubt whether to include or exclude an article, a discussion was held until consensus was reached. First, general and methodological information was extracted from all included articles. Second, the reported findings concerning environmental factors related to older adults’ PA behaviors were extracted. This included the types of environmental factors, how they influenced the participants’ PA behaviors (if available), and illustrating participants’ quotes (if available). An inductive approach was used to analyze the content of the extracted findings.

### Selection criteria

The review included peer-reviewed articles that met the following criteria: (1) participants’ average age was 65 years or older, (2) the study aimed to explore the participants’ experiences of PA and/or the physical environment, (3) the study used qualitative methodologies for data collection and analysis, and (4) the study provided data that can be evaluated. Mixed-methods studies were also included, but only results from the qualitative analyses were included in this review. Studies focusing on unhealthy, overweight, disabled or institutionalized participants were excluded.

### Search strategy

**Figure 1:**
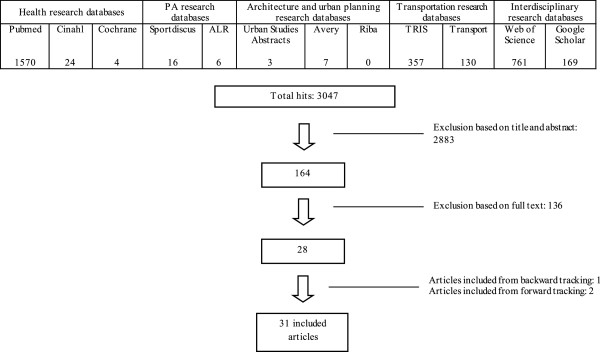
Flow chart of the systematic literature search representing yield and inclusion into the review.

In light of the multidisciplinary nature of our topic, relevant articles were searched in databases of various disciplines, including databases on health (Pubmed, Cinahl, and Cochrane), PA (Sportdiscus and ALR database), architecture and urban planning (Avery, Urban Studies Abstracts, and RIBA), transportation (TRIS and Transport), and interdisciplinary databases (Web of Science and Google Scholar). The search terms included a combination of key words related to the physical environment (e.g., walkability, neighborhood), PA (e.g., leisure activities, active travel), qualitative methodologies (e.g., focus groups, in-depth interviews), and older adults (e.g., elderly, seniors). The full combination of search terms is presented in Figure 1. The retrieved articles were consecutively screened for eligibility by title, abstract, and full text. For all articles included by full text, a back- and forward tracking procedure was performed to identify additional relevant articles. Figure 1 presents a flow chart of our systematic literature search, according to the PRISMA-guidelines [23].

### Data extraction

Systematic data extraction was conducted in order to obtain an overview of the studies’ characteristics and findings. Data extraction started with extracting the general characteristics of the studies: country, setting, sample size, sampling technique and sample characteristics (gender, ethnicity, and socio-economic status), as well as methodological aspects: type of study (intervention-related or pure basic scientific research), methodology, and qualitative data analysis. Secondly, the Results section of each article was read by MM, JVC and RHL independently. Environmental factors potentially related to the participants’ PA behaviors were extracted. At this stage, environmental factors were extracted as they were defined by the authors of the original studies. If available, information on how the environmental factors potentially influenced PA behaviors and illustrating quotes were extracted. In case of any disagreement, a discussion was held until consensus was reached.

### Analysis

Data analysis was conducted independently by MM, JVC, and RHL. Disagreements were resolved by discussions with EC and PP until consensus was reached. An inductive content analysis was performed on the extracted findings following the procedures described by Elo and Kyngas [24]. Firstly, the extracted findings were read thoroughly and notes were made in the text using open coding. Secondly, categorization was applied to merge (1) related environmental factors into *subthemes* and (2) related *subthemes* into *themes*. In a last phase, the so-called abstraction phase, subthemes and themes were named using content-characteristic words. Findings were illustrated using quotes by participants (as reported in the reviewed articles).

To investigate whether the emerging environmental elements (as classified by the authors as “themes”, “subthemes” or “environmental factors”) differed according to the applied qualitative methodology (study aim 3), the identified environmental elements were grouped by study methodologies (i.e. interview versus spatial methods).

## Results

### Characteristics and methodologies of the reviewed studies

The following two subsections address the first aim of this review, which involved describing the characteristics and methodologies of qualitative studies conducted in our research area.

#### General characteristics

A total of thirty-one studies, published between 1996 and 2012, met the inclusion criteria (Table 1). The majority of studies (n = 17) were conducted in North America, followed by eleven studies in Europe, four in Oceania, two in South America, one in Asia, and one was a multi-country study covering all five continents. Most studies (n = 22) were conducted in urban settings, four were conducted in rural settings, and one study was conducted in urban and semi-urban areas. Most studies (n = 28) included men and women, except for three studies that included only women. Eight studies focused on ethnic minorities and five focused on populations of low socio-economic status.

**Table 1:** General characteristics and methodological aspects of the included articles

	**Number of articles**	**Studies reference numbers**
**General characteristics**		
**Country**		
North-America	17	[25-41]
Europe	11	[28,42-51]
Oceania	4	[28,52-54]
South America	2	[28,55]
Asia	1	[28]
**Setting**		
Urban	22	[25-29,31,32,34,36,39,42-49,51,53-55]
Rural	4	[30,33,39,41]
Not reported	6	[35,37,38,40,50,52]
**Gender**		
Female + male	28	[25-32,34-40,42-54]
Female	3	[33,41,55]
**Special populations**		
Ethnic minority	8	[26,33,34,36,38,39,52,55]
Low SES	5	[25,31,47,53,55]
**Methodological aspects**		
**Sampling techniques**		
Purposive	20	[25-27,29-31,33-35,39,42,43,46-50,53-55]
Purposive and convenience	11	[26,28,32,36,37,40,41,45,49,51,52]
**Sample size**		
n ≤ 30	15	[26,27,30,32,38,40,42,45,46,48-50,52,54,55]
30 ‹ n ≤ 60	10	[31,33-36,41,43,44,47,51]
60 ‹ n ≤ 100	4	[25,29,37,53]
n ›100	3	[28,39,46]
**Type of research**		
Pure basic research	24	[25-33,38-41,43-45,47-49,51-55]
Intervention-related research	8	[34-37,39,42,46,50]
**Methodology**		
Pure qualitative	23	[26,28-32,34-41,43,44,47,49-52,54,55]
Mixed-methods	8	[25,27,33,42,45,46,48,53]
**Qualitative data collection method**		
Focus group discussion	20	[25,26,28-34,36-39,41,42,45-47,52,55]
Individual interviews	9	[27,35,40,44,48-50,53,54]
Photo-voice	3	[26,29,32]
Observation	3	[25,31,44]
Walk-along	3	[42,46,51]
Interviews/participant		
Observation		
Virtual reality experiment	1	[43]
**Data analysis method**		
Inductive	17	[26,31,32,35-37,39-42,45,49,51-55]
Deductive	4	[27,28,43,47]
Hybrid*	8	[25,29,33,34,38,44,48,50]
Not reported	2	[30,46]
**Qualitative analysis software used**		
Atlas/ti	3	[32,39,44]
Nvivo	3	[47,48,51]
NUD*IST	2	[33,35]
N4	1	[52]
Not reported	22	[25-31,34,36-38,40-43,45,46,49,50,53-55]
**Data analysis validation**		
Member checking	2	[25,31]
None reported	29	[26-30,32-55]

*Combination of inductive and deductive.

#### Methodological aspects

From the 31 studies, 23 exclusively used qualitative methods and eight studies combined qualitative and quantitative methods (mixed-methods studies). Overall, 29 studies used indoor interviews; 20 studies used focus groups and 9 studies used individual interviews. All individual and focus group interviews were mediated according to pre-determined guidelines (including instructions, questions, prompts etc.), which mostly focused on either the physical environment or PA. Only four interview studies used guidelines regarding both the physical environment and PA. Ten studies employed spatial qualitative methods, of which nine combined indoor individual or focus groups interviews with spatial qualitative methods. Of these ten studies, three studies used photo-voice methodology, in which focus group participants discussed environmental factors depicted in photographs they took prior to the interview. In three other studies the researchers performed on-site observations in the study area before or after indoor interviews were held. Three studies included walk-along interviews, which consisted of an interview during a walk along a route usually chosen by the participant from his/her home to a specific destination. One study used virtual routes to explore older adults’ perceptions of pedestrian routes.

From the 31 studies, 17 employed an inductive analysis, four a deductive analysis and eight a hybrid analysis. The remaining two studies did not mention a data analysis approach. In the inductive analyses, the researchers derived (sub)themes directly from the qualitative information gathered from the informants. Deductive analysis studies classified the environmental attributes mentioned by the informants according to pre-existing categories of environmental features studied previously in the literature. Several analytical techniques were mentioned, such as: content analysis [28], grounded theory [44], framework analysis [30], and successive approximation [29]. Two studies used member checking to validate the researchers’ interpretations against the participants’ meanings.

### Environmental themes identified in the reviewed studies

Table 2 provides an overview of the themes, subthemes, and environmental factors that were identified in the reviewed articles. This addresses the second aim of this review, which was to identify recurring physical environmental themes and factors possibly related to older adults’ PA behaviors. In order to illustrate the findings, selected quotes of participants’ are presented in Table 2. The following five environmental themes emerged from the data: (1) pedestrian infrastructure, (2) safety, (3) access to facilities, (4) aesthetics, and (5) environmental conditions. Corresponding subthemes and environmental factors are described in detail below.

**Table 2:** Themes, subthemes, environmental factors and illustrating quotes reported in the reviewed studies

**Themes**	**Subthemes**	**Environmental factors**	**Informants’ quotes**
**Pedestrian infrastructure**	**Sidewalks’ characteristics**	● **Sidewalks’ presence and continuity** (i.e., lack of sidewalks, abrupt ending of sidewalks, integration of pedestrian routes, continuous sidewalks).	*“Since I have started to use my rollator, I immediately noticed how high the curbs were as well as all other types of barriers.”*[42]
		● **Sidewalks’ quality and maintenance** (i.e., poorly laid and maintained paving, poor snow clearance, icy sidewalks, sidewalks width, smooth surfaces).	*“And the high curbs, so if we are going to a certain place we have got to say ‘now we have got to go along there and there’s a low curb there, and go down here, but I have got to cross there and move along there’. You can’t just go from A to B.”*[27]
		● **Sidewalks’ slopes and curbs** (i.e., absence of steep gradients; cracked, uneven, steeply sloped, or high curbs, railings along steep sidewalks and stairs, strategically placed curb cuts).	
		● **Temporary obstacles on sidewalks** (i.e., dog leashes, carts/fallen fruit on sidewalks, cars/bicyles parked on sidewalks).	*“You cannot get to the stop half the time because it is icy and if you walk down the road, you cannot climb up over the bank to get to where the bus stop is, because it is all filled up with ice. I find anywhere in the wintertime around here, any bus stop, they are not cleared out.”*[31]
	**Separation between pedestrians and non-motorized transport**	**● Cyclists on sidewalks**	*“Recently, they have renewed the sidewalks over here. The situation was really bad. Now it’s better with those red tiles marking the cycling path. Cyclists know where to cycle now. Before, everything was mixed up.”*[51]
		● **Skateboarders and roller-bladers on sidewalks**	
**Safety**	**Crime-related safety**	**● Lack of street lightning**	*“Poor street lighting would prevent me from walking in the evening. Overgrown bushes, shrubs… sometimes you have abandoned homes, and the shrubbery has gotten out of control.”*[43]
		● **Upkeep** (i.e., vacant houses, overgrown lots, vandalism).	
		● **Other people** (i.e., few people walking around, large crowds, intimidating people, friendly and socially responsible other people).	*“The only problem is that around six or seven p.m., the city center is dead. So we won’t go out anymore. During summer there are a lot of people on the terraces. But during this weather, it is dead at six or seven pm. Traffic is not allowed anymore, so people don’t come. I’m always in a hurry to get home because there’s so little movement out here.”*[51]
		● **Presence of authorized personnel** (i.e., slow or inappropriate police, worrying presence of police, senior patrol, police or security, staff in public facilities).	
	**Traffic-related safety**	● **Zebra-crossing characteristic** (i.e., unclear indication, long distances between crossings, inadequate signal times (too short), long crossing distances).	*“I feel that we need… something… because in the winter you don’t want to hurry across the street when you see there’s no traffic… It’s fine once you get to the crossing, but there may be long, long, long distances.”*[32]
		● **Reckless driver’s behavior** (i.e., impatience, speeding, use of cell phones).	
**Access to facilities**	**Access to exercise opportunities**	● **Access to recreational facilities** (i.e., lack of exercise facilities, facilities for older adults located to far from home, lack of transportation to recreational facilities, costs of recreational facilities).	*“They’re not including us! They’re more concerned about the young people, what they’ve got. They’ve got skate parks and all sorts of things they’re planning for them, but they’re not planning anything for us.”*[53]
			*“I would like to see a gym that I can afford. They have gyms, but I can’t afford to join one.”*[39]
		● **Access to senior oriented group activities** (i.e., leisure provision primarily designed for younger people; feeling uncomfortable and unsafe without instructions, age-appropriate forms of leisure provision; group activities designed for seniors; indoor gym, pool, and dedicated buildings for seniors).	*“We can go walking through the woods there and there’s a jolly good hour walk around through the woods up to the top onto the park and down the road and back again… that’s quite a good run.”*[48]
		● **Access to green open space** (i.e., isolated trails, nearby parks and woods).	
	**Access to daily destinations**	● **Access to daily destinations** (i.e., shops and services, senior center).	*“The grocery store was just across the street. The bank, the liquor store, the hairdresser, and everybody just walked and met everyone. . . . It was quite pleasant. Today, we have to get into our cars. So, that has really changed.”*[31]
			*“Providing transport to pick up older people from various homes would be a good help. I think that some older people don’t participate because they don’t have transport.”*[52]
		● **Access to public transit** (i.e., bus-stop characteristics: long distances, shelter, senior oriented bus-service).	*“I used to be able to walk downtown no problem, but as you get old, you slow down, so now I gratefully have my senior’s pass and I use it.”*[31]
	**Access to rest areas**	● **Access to benches** (i.e., presence of benches usability of benches).	*“If [older people] are out round to the shops, or the community center here, they could always walk back and sit in there in the summer for half an hour if you like and have a rest. You have always got to remember that the older ones like us, you can get tired.”*[44]
		● **Access to public washrooms** (i.e., presence of clean washrooms nearby daily destinations).	*“I’d probably put up a seat or two to sit on the way…I mean even going along, there’s a post box along on the main road and I cut through – um – the social club, but I nearly always sit down in the bus shelter on the way.”*[48]
**Aesthetics**	**Buildings and steetscape**	● **Private property** – challenges (i.e., signs of neglect), opportunities (i.e., well-maintained private property).	*“Here it’s getting more interesting to walk; you have the park on the one side and some very beautiful houses on the other side. These are all from the beginning of the last century and I really like some of them.”*[51]
		● **Public realm** – opportunities (i.e., attractive streetscapes, historical buildings).	
	**Natural scenery**	**● Presence of greenery**	
		**● Presence of water**	
**Environmental conditions**	**Weather**	**● Cold weather**	
		**● Hot weather**	
		**● Warm weather**	
	**Environmental quality**	**● High environmental quality**	
		**● Pollution**	

#### Pedestrian infrastructure

The theme pedestrian infrastructure included two subthemes: (1) sidewalk characteristics, and (2) separation between pedestrians and other non-motorized transport.

Participants mentioned several sidewalk characteristics that may facilitate/hinder walking, such as: sidewalk presence and continuity, sidewalk quality and maintenance, slopes and curbs, and temporary obstacles on sidewalks. Concerning sidewalk presence and continuity, participants preferred streets with sidewalks over streets that lacked sidewalks. Furthermore, they disliked abrupt endings of sidewalks which forced them to walk on the street or a parking lot. When sidewalks were present, older participants did not like the presence of a steep gradient. In the presence of hills or stairs, they liked the presence of handrails. Furthermore, they disliked cracked, uneven, steeply sloped, or high curbs. Some participants complained about curbs that were impossible to negotiate with a walker and desired strategically placed curb cuts (e.g., lower curbs at zebra crossings and higher curbs at bus stops). Concerning sidewalk quality and maintenance, participants discussed issues such as sidewalk width, smoothness of sidewalk surfaces, holes, and cracks. Specifically, weather-related sidewalk maintenance aimed at removing snow and ice emerged as an important factor, reflecting older adults’ fear of falls. In addition, temporary obstacles on sidewalks were mentioned as a barrier for walking. Examples of such obstacles were dog leashes (especially for those with impaired sight), shopping carts, fallen fruit, and parked cars or bicycles.

The subtheme “separation between pedestrians and other non-motorized transport” concerned sidewalks being used by cyclists and other non-motorized transport (e.g., rollerblades, skateboards). This was mentioned as a barrier for walking, reflecting older adults’ fear of being hit or injured. Consequently, a clear separation between sidewalks and cycling paths emerged as conducive for walking.

#### Safety

The theme “safety” included two subthemes: (1) crime-related safety and (2) traffic-related safety.

Participants stated that fear of crime was higher in the absence of street lighting. Participants were also more fearful in areas that were not well-kept. They disliked vacant houses, overgrown lots and vandalism (e.g., graffiti, littering, and sabotage of benches). Desolate streets were also mentioned as decreasing the sense of crime-related safety. The presence of people in the street was mentioned as both increasing and decreasing the sense of personal safety depending on the type of people. The presence of families with children, friendly, smiling, and familiar people, socially responsible residents, or people walking, biking or jogging were considered to improve crime-related safety. On the other hand, large crowds, criminality, and the presence of intimidating groups of youths, beggars, immigrants, and homeless people were perceived as decreasing crime-related safety. In the same manner, the presence of police and other law-enforcement staff was mentioned as having both positive and negative effects on crime-related safety. Positive effects were attributed to the presence of senior patrol, police or security personnel, and to the presence of staff in public facilities. Negative effects were attributed to slow or inappropriate police response to neighborhood crime and to the worrying presence of police.

Within the subtheme “traffic-related safety”, two different environmental factors were identified: zebra-crossing characteristics and reckless driver’s behaviors. Zebra-crossing characteristics emerged as a major issue. Participants mentioned several zebra-crossing’s attributes that made it difficult and unsafe to cross roads, such as: unclear indication of pedestrian crossing, long crossing distances across multiple traffic lanes and inadequate signal times (e.g., too short green crossing phases). Interestingly, long distances between regulated pedestrian crossings were mentioned as a reason for ignoring red traffic lights. Other traffic-related issues concerned reckless driving behaviors, including speeding, impatient drivers, and drivers distracted by phoning while driving.

#### Access to facilities

The theme “access to facilities” was subdivided into (1) access to exercise opportunities, (2) access to daily destinations, and (3) access to rest areas.

For access to exercise opportunities, it was generally argued that there are not enough recreational facilities for older adults. Additional problems were having existing facilities located too far from home, the lack of transportation to those facilities, and the high costs to use the facilities. Age-appropriate provision and senior-oriented group activities emerged as essential. Moreover, some informants mentioned that leisure provision is primarily designed for younger people and raised the need for group activities designed for seniors. Following facilities were preferred: indoor gyms, indoor pools, and buildings dedicated to older adults. Informants also mentioned feeling uncomfortable and unsafe exercising in recreational facilities without instructions. Green open space was also mentioned as an inviting setting for PA. However, participants did not like to use isolated trails in wooded areas with poor visibility.

The subtheme “access to daily destinations” consisted of two environmental factors: access to daily destinations, and access to public transit. Participants described the importance of having access to various daily destinations to stimulate their walking. They mentioned access to general shops and services, such as grocery stores, libraries, mailboxes, newspaper-boxes, post offices, but also mentioned specific senior-oriented amenities (e.g., senior centers). Participants also liked to have easy access to public transit and disliked long distances to bus stops. Sheltered bus stops were mentioned as a positive feature. Additionally, senior-oriented bus services were discussed, including pick-up services at home and community buses serving community centers or retirement homes. The need for a good bus service was accentuated in light of age-related physical changes that shorten the distance older adults are capable of walking, and made them more reliant on public transit.

The third subtheme was access to rest areas, including access to benches and public washrooms. Various aspects concerning the presence of benches emerged, including distance between seating areas along walking routes and in hilly areas. Also, the usability of seating areas was mentioned referring to designing benches that are easy to sit on and the importance of sheltered benches, especially during the winter. The need for seating areas was accentuated in light of older adults’ physical limitations and their increased need for rest. Access to public washrooms also emerged as an important issue, including the presence of clean washrooms in public areas close to daily destinations.

#### Aesthetics

The theme “aesthetics” included the subthemes: (1) buildings and streetscape, and (2) natural scenery.

Concerning buildings and streetscape, characteristics of private as well as public properties emerged as important factors. Neglected areas (e.g., vacant houses, overgrown lots, fallen trees or branches, and weeds overgrowing sidewalks) discouraged walking and PA. In contrast, participants liked streets inhabited by socially responsible residents who took care of their homes and gardens. Participants liked the presence of historical buildings and attractive streetscapes, including buildings with personal significance, statues, distinctive buildings, buildings at a human scale, or architectural variation between houses. In addition to attractive buildings, participants also enjoyed the presence of nature, including the presence of trees and water.

#### Environmental conditions

Environmental conditions were subdivided into: (1) weather and (2) environmental quality.

Participants preferred warm rather than cold weather. They liked the pleasant warm weather during spring, but disliked cold temperatures, wind, ice, snow, rain, and early darkness. However, hot weather was disliked as well; participants mentioned high temperature, humidity and strong sun radiation as barriers to walking and PA.

Concerning environmental quality, participants preferred environments with high environmental quality that are quiet and peaceful and provide fresh air. On the other hand, they disliked polluted areas with high levels of traffic exhaust fumes and noise.

### Comparison of the emerging themes and factors according to the qualitative method used

Table 3 presents the themes (along with a few subthemes and environmental factors) that were identified in the various studies according to the qualitative methodologies used. This addresses the third aim of this review. We compared results obtained in studies using interviews (individual or focus group) versus studies using spatial qualitative methods (photo-voice, observations, walk-along interviews, and virtual reality experiments). As was shown in Table 1, most studies using spatial qualitative methods combined them with interviews and/or focus groups.

**Table 3:** Themes, subthemes and environmental factors identified in the reviewed studies by research methods

**Themes, subthemes and environmental factors**	**Number of studies using**		**Studies**
	**Interviews (n = 21)**	**Qualitative spatial methods (n = 10)**	
Pedestrian infrastructure	22	10	[25,26,29-33,35,39,42-46,48,51,53]
Separation between pedestrians and other non-motorized transport^1^	6	5	[25,31,36,42,45,46,51]
Weather-related sidewalk maintenance^1^	5	4	[25,26,32,42,45]
Crime-related safety^2^	14	6	[26,29,30,32,33,36,37,42,43,45,47,48,51-53,55]
Traffic-related safety^2^	17	10	[25,26,29-32,38,42-46,49,51-53]
Access to facilities	9	8	[25,26,29,31,32,43,44,47,48,51,52]
Access to exercise opportunities^3^	15	2	[30,31,33,34,38,39,41,42,47,48,50,52-55]
Green open spaces^3^	11	9	[25,26,29,31,42-44,46-49,51,53]
Public transit^3^	14	6	[25,29,30,32,34,39,40,44-46,48,50-52,54]
Rest areas^3^	9	7	[26,29,31,32,44-46,48,50,51]
Aesthetics	9	8	[25,26,29,32,43,44,46-48,51,53]
Weather^4^	8	2	[26,30,31,37,39,41,47,55]
Environmental quality^4^	6	4	[26,31,44,46,48,53]

^1^subthemes and environmental factors categorized under the theme “pedestrian infrastructure”; ^2^subthemes categorized under the theme “safety”; ^3^subthemes and environmental factors categorized under the theme “access to amenities”; ^4^subthemes and environmental factors categorized under the theme “environmental conditions”.

Frequency of emergence of certain environmental factors and (sub)themes appeared to differ between indoor interview and spatial qualitative methods. Several factors and (sub)themes tended to be reported more frequently in spatial compared to indoor interview methods. These included: separation between pedestrians and other non-motorized transport, weather-related sidewalk maintenance, access to facilities, green open spaces and rest areas, aesthetics and environmental quality. Two themes were reported more frequently in studies using interviews compared to studies using spatial methods: weather and access to exercise opportunities. No other discrepancies were observed between studies using indoor interviews and spatial qualitative methods.

## Discussion

The current study aimed to systematically review the qualitative literature on the physical environment and PA among older adults. We retrieved 31 relevant articles, which varied considerably in setting and methodology. Five environmental themes were identified as potentially influencing older adults’ PA: pedestrian infrastructure, safety, access to facilities, aesthetics, and environmental conditions. Additionally, we obtained detailed in-depth information on how and why the emerging environmental factors influence older adults’ PA.

All included studies described the importance of pedestrian infrastructure. However, in a systematic review of quantitative studies results concerning walking facilities were found to be inconsistent, with the majority of studies yielding a non-significant relationship with PA behaviors [15]. Our findings showed that a variety of sidewalk characteristics might influence their use. For example, participants discussed not only the presence of sidewalks, but also their continuity, slopes and curbs, maintenance, separation from cyclists, etc. Hence, there are many factors influencing the use of sidewalks, which are likely not captured comprehensively in questionnaires used in quantitative studies.

Safety issues, crime- as well as traffic-related, also emerged in almost all qualitative studies as influencing older adults’ PA. In contrast, findings from quantitative studies are equivocal [15]. In the current review, crime- and traffic-related safety emerged as multidimensional constructs, including physical as well as social components. This supports previous calls [56,57] for more comprehensive measures to assess perceived crime- and traffic-related safety.

Several quantitative studies have consistently reported positive relationships between objective and perceived access to destinations and older adults’ PA behaviors [12-14,58]. Similarly, older adults in the qualitative studies mentioned easy access to shops, services and senior centers as facilitators of walking and PA. Moreover, they also expressed the need for easy access to public transit. Concerning recreational activity, issues related to the accessibility (e.g., too far away, no transportation) and costs of exercise facilities were frequently noted in the reviewed articles. Participants also expressed a need for age-appropriate forms of PA, including group activities and supervision. Consequently, when studying the relationships between access to PA facilities and older adults’ PA, it might not be sufficient to study merely the presence of general exercise facilities. Our findings suggest that more detailed information about the specific programs offered at the facilities (e.g. provision of age-appropriate group activities) should be included in future studies. Our findings also indicated that informal settings, such as parks, can stimulate older adults’ PA. However, participants were averse to isolated trails in wooded areas with low visibility, possibly due to increased fear of crime [59]. Furthermore, our findings revealed that not only access to different types of destinations was important, but also the presence of resting areas at and on the routes to these destination. The presence of benches, preferably sheltered, was stated to facilitate walking as they provide the opportunity to rest, especially for those with decreased functional capacity. However, two previous quantitative studies reported no significant relationships for transportation walking with objective [60] and perceived presence of benches [58]. Our qualitative findings might explain this contradiction, as it was shown that not merely the presence of benches might influence older adults’ PA, but also their usability in terms of design (benches easy to sit on for older adults) and accessibility in winter (sheltered benches). These specific details are unique to older adults and reflect the ability of qualitative methods to reveal in-depth information on what, how, and why environmental factors are related to older adults’ PA. Next to benches, the current study also found that the presence of clean washrooms was a potential facilitator of older adults’ PA.

Our findings suggest that aesthetically appealing places, which are well-maintained and include attractive buildings and natural elements, facilitate older adults’ PA. Neglected areas might not only discourage PA for aesthetic reasons, they might also increase fear from crime and, therefore, inhibit older adults’ PA [56]. However, the majority of previous quantitative studies reported no relationship between aesthetics and older adults’ PA behaviors [15]. Possibly, as was proposed by Alfonzo [8], the aesthetic appeal of a place might be a less important theme when compared to pedestrian infrastructure, access to facilities, or safety, and might only come into play when the environment is already generally favorable for PA (e.g., safe places with high-quality sidewalks and easy access to facilities). Participants also preferred unpolluted areas that provide fresh air over areas with car exhaust fumes and traffic noise. Furthermore, the participants’ statements reflected seasonal effects. Participants preferred the comfortable warmth of spring time as opposed to the heat of the summer or the cold, snow, ice, and darkness of winter.

Findings of the reviewed qualitative studies add depth and detail to the results of previous quantitative research. Our findings suggest that a more comprehensive assessment of certain environmental factors in quantitative studies might lead to a more accurate understanding of environment-PA relationships in older adults. The qualitative studies highlight the importance of micro-scale environmental characteristics (e.g., quality of sidewalk and presence of benches), which might be especially relevant for older adults’ PA, but which have not been linked consistently to older adults’ PA in previous quantitative studies. However, most studies included in our review employed focus groups and/or individual interviews, while only a few studies employed spatial qualitative methods. These spatial qualitative methods are especially useful in understanding the physical environment from the informants’ perspectives. This is particularly essential among older adults, who develop unique environmental needs due to age-related changes (decreased functional capacity, impaired sight or hearing, etc.). Although our comparison between themes revealed by interview versus spatial methods was rather preliminary, it did illustrate the added value of spatial qualitative methods. It showed that themes, which reflected unique environmental needs of older adults (e.g. access to resting areas), were frequently reported in studies using spatial methods. Combining individual or focus group interviews with spatial methods in future research can add depth to our understandings of PA-environment relationships by connecting specific objective environmental attributes to the subjective experiences of informants.

In the reviewed studies, most of the individual and focus group interviews were conducted according to predetermined guidelines that focused on either the physical environment or PA, while only a few focused on both the physical environment and PA. Consequently, the findings of these studies focused primarily on the informant’s views on either the physical environment (e.g., perceived walkability) or PA (e.g., PA barriers and facilitators). Future qualitative studies should include guidelines that include both descriptions of the physical environment and PA.

Some limitations of the current review should be acknowledged. First, we made no distinction between different PA domains. There are several reasons for this; many different types of physical activities were studied in the reviewed articles, some articles did not explicitly define the physical activities targeted, and in the Results sections findings for different physical activities were often mixed up. Hence, future qualitative studies should explicitly define which PA behavior(s) they targeted. This also requires that qualitative researchers should provide clear instructions to their participants regarding which activities to consider during data collection. Secondly, only two of the included articles used member checking as a way to validate the researchers’ interpretation of data against the participants’ intended meanings. The use of member checking should be encouraged in future qualitative studies. A primary strength of the current study is the comprehensive search in multiple databases reflecting the multidisciplinary nature of the topic. Moreover, we used the summarized qualitative information to complement and explain inconsistencies observed in previous quantitative research.

To conclude, this review provided an overview of the characteristics and findings of qualitative studies in the research area of environment-PA relationships in older adults. Additionally, we observed some discrepancies in emerging environmental factors and themes between interview-based and spatial qualitative methodologies. Based upon the reviewed qualitative studies, in order to promote PA among older adults, environments should (1) provide high-quality pedestrian infrastructure, (2) be safe from crime and traffic, (3) provide easy access to exercise opportunities, daily destinations and rest areas, (4) be aesthetically appealing, and (5) provide pleasant environmental conditions. Our findings showed that qualitative research can provide in-depth information on not only which, but also how and why environmental factors influence older adults’ PA. It was shown that it is not just the mere presence of an environmental attribute (e.g. a sidewalk), but also its quality (e.g. continuity, evenness, maintenance, separation), that should be taken into account when designing environments that aim to stimulate PA among older adults. This finding might also explain previously observed inconsistencies between quantitative studies. Hence, future quantitative studies should not only take into account the presence of certain environmental attributes but also their quality. From a methodological perspective, given the interdisciplinary nature of our topic, including both the physical environment and PA in interview guidelines and combining interviews with more spatially-oriented methods may provide a fuller and more nuanced description of environment-PA relationships. Good examples of such interdisciplinary collaborations can already be found in quantitative studies, which combine geographic measurements (e.g., GIS and environmental audits) with health data (e.g., accelerometer-derived and self-reported PA and functional capacity) [13,14,61,62]. Therefore, mixed-methods studies, including both quantitative and qualitative methods, may provide a good platform for interdisciplinary collaborations that can result in establishing quantitative relationships complemented with in-depth qualitative information.

## Competing interests

The authors declare that they have no competing interests.

## Authors’ contributions

All authors contributed to the design of different parts of the study. MM was responsible for the conceptualization and design of the manuscript, wrote a substantial part of the manuscript and led the writing team. JVC equally contributed to the work by reviewing and analyzing half of the articles and participating in writing the manuscript. RHL made substantial contributions to the design, acquisition of data, and summarization of findings. EC, PP and BD revised the entire manuscript and made important contributions in various sections. All authors read and approved the final manuscript.
